# A Promising Tool to Assess Long Term Public Health Effects of Natural Disasters: Combining Routine Health Survey Data and Geographic Information Systems to Assess Stunting after the 2001 Earthquake in Peru

**DOI:** 10.1371/journal.pone.0130889

**Published:** 2015-06-19

**Authors:** Henny Rydberg, Gaetano Marrone, Susanne Strömdahl, Johan von Schreeb

**Affiliations:** Department of Public Health Sciences, Karolinska Institutet, Stockholm, Sweden; Örebro University, SWEDEN

## Abstract

**Background:**

Research on long-term health effects of earthquakes is scarce, especially in low- and middle-income countries, which are disproportionately affected by disasters. To date, progress in this area has been hampered by the lack of tools to accurately measure these effects. Here, we explored whether long-term public health effects of earthquakes can be assessed using a combination of readily available data sources on public health and geographic distribution of seismic activity.

**Methods:**

We used childhood stunting as a proxy for public health effects. Data on stunting were attained from Demographic and Health Surveys. Earthquake data were obtained from U.S. Geological Survey’s ShakeMaps, geographic information system-based maps that divide earthquake affected areas into different shaking intensity zones. We combined these two data sources to categorize the surveyed children into different earthquake exposure groups, based on how much their area of residence was affected by the earthquake. We assessed the feasibility of the approach using a real earthquake case – an 8.4 magnitude earthquake that hit southern Peru in 2001.

**Results and conclusions:**

Our results indicate that the combination of health survey data and disaster data may offer a readily accessible and accurate method for determining the long-term public health consequences of a natural disaster. Our work allowed us to make pre- and post- earthquake comparisons of stunting, an important indicator of the well-being of a society, as well as comparisons between populations with different levels of exposure to the earthquake. Furthermore, the detailed GIS based data provided a precise and objective definition of earthquake exposure. Our approach should be considered in future public health and disaster research exploring the long-term effects of earthquakes and potentially other natural disasters.

## Introduction

Earthquakes are natural disasters that impact health and society. During the past two decades alone, earthquakes have caused nearly one million deaths globally [1]. Other severe consequences of earthquakes include injuries, loss of assets and livelihood, as well as disruption of infrastructure and societal functions [2].

Research into the health effects of earthquakes has overwhelmingly focused on immediate consequences, such as on injury and mortality. Furthermore, most studies concentrate on high-income settings [3], although the risk of death and damage from earthquakes is significantly higher in low and middle-income countries [4]. It has also been reported that in these lower income settings, sudden shocks have greater long-term negative impacts on society and development than in higher income regions [5,6]. However, such prolonged effects are difficult to define and quantify and, thus, have not been well studied.

One way to quantify long-term effects of earthquakes could be to monitor changes in important public health indicators. Such indicators often reflect living conditions, socioeconomic status and other factors that affect the general well-being of a population, and that can be affected by an earthquake. Furthermore, such indicators are often routinely collected in national health surveys. For example, Zolala et al considered maternal mortality a useful indicator to measure long-term health effects of the 2003 Bam earthquake, since maternal deaths were likely to be affected by the earthquake’s impact on women’s living conditions and access to health care [7]. Childhood stunting is another important public health indicator. Stunting correlates with the degree of food insecurity, poverty and disease, and could thus indicate long term effects on public health [8,9]. Previous studies have found high levels of stunting in areas affected by earthquakes [10]. Data on childhood stunting is regularly collected in the Demographic and Health Surveys (DHS), which is an important source of health information in low- and middle-income countries. In most recent DHS surveys, the surveyed clusters are geo-referenced, which may allow levels of stunting to be tracked according to the geographical distribution of a disaster such as an earthquake [11].

Data on the distribution of earthquakes and other natural disasters have also recently become more accessible due to the increasing use of geographic Information Systems (GIS) [12]. For example, the U.S Geological Survey’s (USGS) ShakeMap atlas is a rich source of GIS data for earthquakes [13]. ShakeMaps divide earthquake-affected areas into different zones of seismic intensity. Seismic intensity represents the level of ground shaking in a given location and is closely related to the level of human impact and destruction [14]. Combined with data on population distribution, the population affected by the disaster can be estimated with high precision [15].

In this study we explored whether long-term public health effects of earthquakes can be assessed using a combination of readily available data sources on public health and geographic distribution of seismic activity.

## Methods

### Selection of earthquake case

We tested the approach using a real case—an earthquake that hit southern Peru on June 23rd 2001. Three criteria were used to select the case. First, DHS data had to be available both before and after the earthquake, with significant time (at least two years) elapsing between the earthquake and the last measuring-point. Second, the ShakeMap atlas had to include GIS-based intensity data for that specific earthquake. Finally, the clusters surveyed in DHS had to be geo-referenced. The latter two criteria were deemed necessary to geographically link households to the corresponding earthquake intensity level.

### Peru and the earthquake

Peru is a middle income country [16]. Rapid economic growth over recent years has yielded a decline in poverty, but economic disparities within the country remain high [17,18]. The majority, 91 percent, of the population is ethnic Spanish, followed by the indigenous ethnic group Quechua, which constitutes 7 percent of the population [19]. Stunting remains a significant health problem in Peru, especially in poor highland regions. In 2006, the prevalence ranged from 9% in the richest region, to 57% in the poorest [20]. Stunting prevalence is higher among indigenous groups and in rural areas [21].

**Fig 1:**
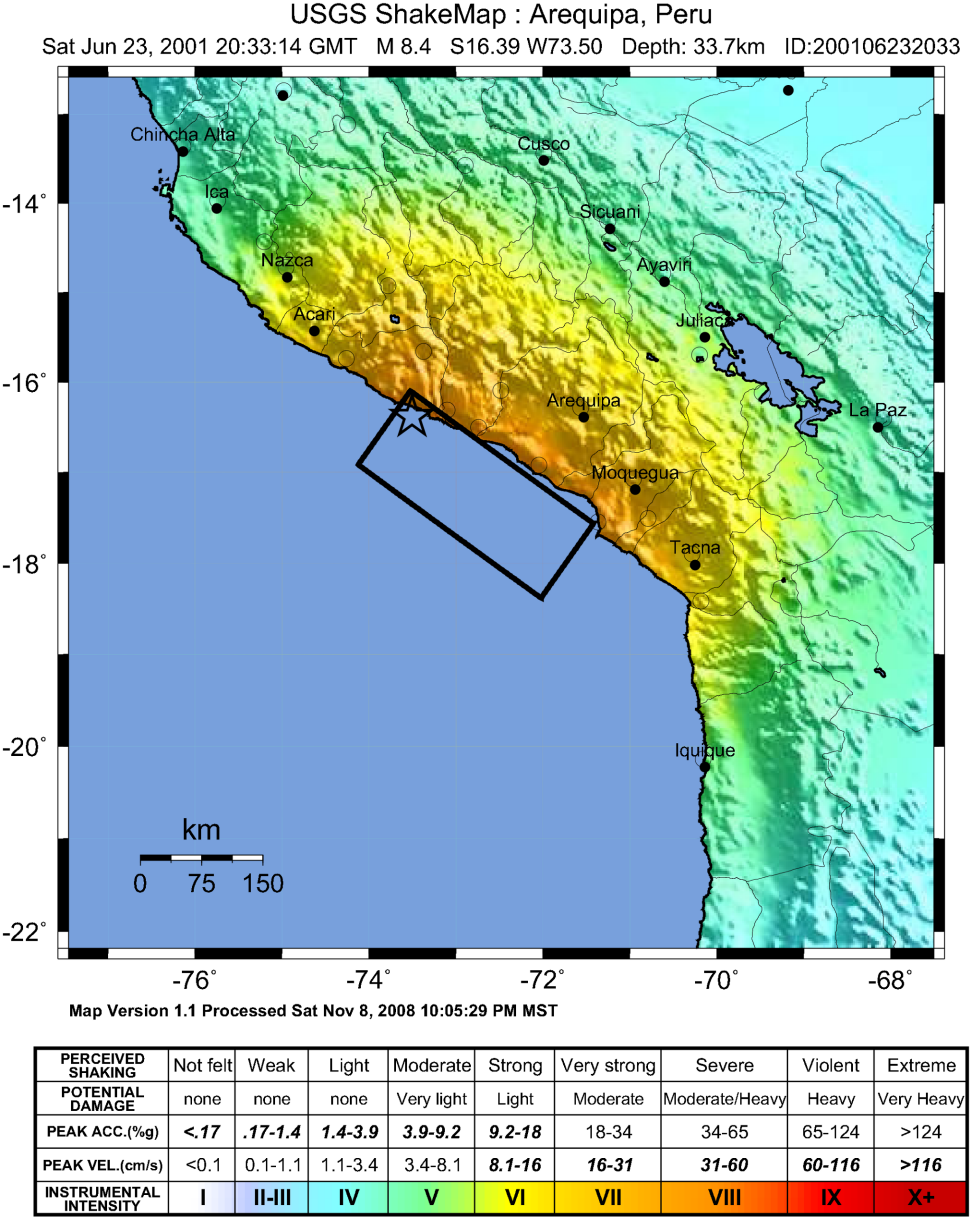
ShakeMap image of the 2001 southern Peru earthquake [21].

Peru lies on an area of intense seismic activity and is frequently affected by earthquakes. The earthquake investigated in this study struck outside the southern coast and measured 8.4 on the Richter scale (see Fig 1). Over 300,000 people were affected, and 145 were killed [1]. The earthquake caused devastation in the affected regions, especially to adobe built houses [22]. According to the EM-DAT disaster database, four administrative regions were affected—Arequipa, Moquegua, Tacna, and Ayacucho [1].

### Data sources

Two DHS surveys were considered for our study: one *standard DHS* [23] and one *continuous DHS* [19,24]. The standard DHS was conducted in 2000 (one year before the earthquake) and sampled 1,414 randomly selected clusters in different parts of Peru. In the continuous DHS, five equally-sized subsamples of these 1,414 clusters were surveyed in five separate rounds over five consecutive years (2004–2008). For our study, this implied that most clusters surveyed before the earthquake were surveyed again a few years after. Anthropometric indicators (weight and height measurements) were collected for children under five years of age in the surveyed households. In the continuous DHS survey, anthropometric data was collected in the 2005, 2007, and 2008 rounds of the survey. The total response rate was 95% in the 2000 standard survey [23] and 98% in the continuous survey [19]. About 90% of children under five years of age were measured for height in both surveys [19,23,24].

The surveyed clusters were geo-referenced, and data files with the geographical location (GPS) of the clusters were provided in addition to the survey data sets. To assure respondents’ confidentiality, the GPS positions were randomly displaced up to two kilometres in urban areas and ten kilometres in rural areas [11].

In USGS ShakeMaps, seismic intensity levels are derived instrumentally, by combining instrumental measurements with information about local seismic conditions and the earthquake source [25]. Seismic intensity is expressed in the Modified Mercalli Intensity Scale (MMI), which ranges from I (shaking not felt, no potential damage) to X (extreme perceived shaking, very heavy potential damage). The 2001 Peru earthquake produced intensities up to VIII MMI (see Fig 1).

We used a GIS software program (ArcGIS version 10.1) to link each surveyed cluster to the corresponding MMI value in the area. The MMI zone map and the cluster GPS-point map were overlaid and joined using the software’s spatial join tool. This created a data set that contained the unique cluster numbers and the corresponding MMI values. This data set was subsequently merged with the full DHS data set, using the cluster number as the common variable.

### Ethical consideration

This study was based on secondary analysis, using data from publically available databases (DHS and USGS Shakemap archive). The DHS surveys were approved by the the Peruvian National Institute for Statistics (INEI) and the ICF Macro at Calverton in the USA. Data were collected with informed consent and anonymity of respondents was assured. Geographical data did not disclose the location of individual households [11].

### Variables

Stunting was defined as a height-for-age more than two standard deviations below the median of the World Health Organization (WHO) growth reference population [26]. Biologically implausible z-scores (z-scores below -6 or above 6, according to WHO Child Growths Standard recommendations [26]) were recoded as missing data.

The MMI values were categorized into three intensity level zones. MMI values I through IV, were not portrayed on the intensity map and were therefore collapsed into the low intensity zone. The remaining observations were divided into medium and high intensity zone by the median MMI value. As a result, the medium intensity zone included MMI value V and the high exposure zone values VI through VIII.

Children were categorized into pre- and post-earthquake groups according to their year of birth. This categorization minimized the risk of including prevalent cases of stunting in the post-earthquake group, i.e. children who were already stunted before the earthquake. Children born before the earthquake (1995–2000) comprised the pre-earthquake reference group. The two post-earthquake groups consisted of children born shortly after (2001–2003), and a few years after (2004–2008) the earthquake respectively. The cut-offs between these two groups was defined by the median birth year.

Other covariates collected from the DHS survey data sets included age of the child (0, 1, 2, 3 and 4 years of age), motherhe child (0,al level (no education, primary, secondary, higher), and availability of household electricity (available, not available). The latter two were considered proxies for socioeconomic status.

### Statistical analysis

We first conducted descriptive analysis by comparing the prevalence of stunting between the birth year groups and, in turn, comparing these differences between the intensity zones. The descriptive analysis was restricted to children over two years of age, as few incident cases occur after this age [27]. Multilevel logistic regression analysis was subsequently performed to account for potential cluster effects (within clusters and within families). We defined three separate regression models—one for each intensity zone—with birth year group as independent variable and stunting as dependent variable. Model 1 adjusted for age of the child whereas Model 2, in addition, adjusted for the level of the motherof for istic regavailability of household electricity, both proxy indicators for socioeconomic status. All statistical analyses were performed in STATA, version 12.0. P-values less than 0.05 were considered statistically significant.

## Results

**Fig 2:**
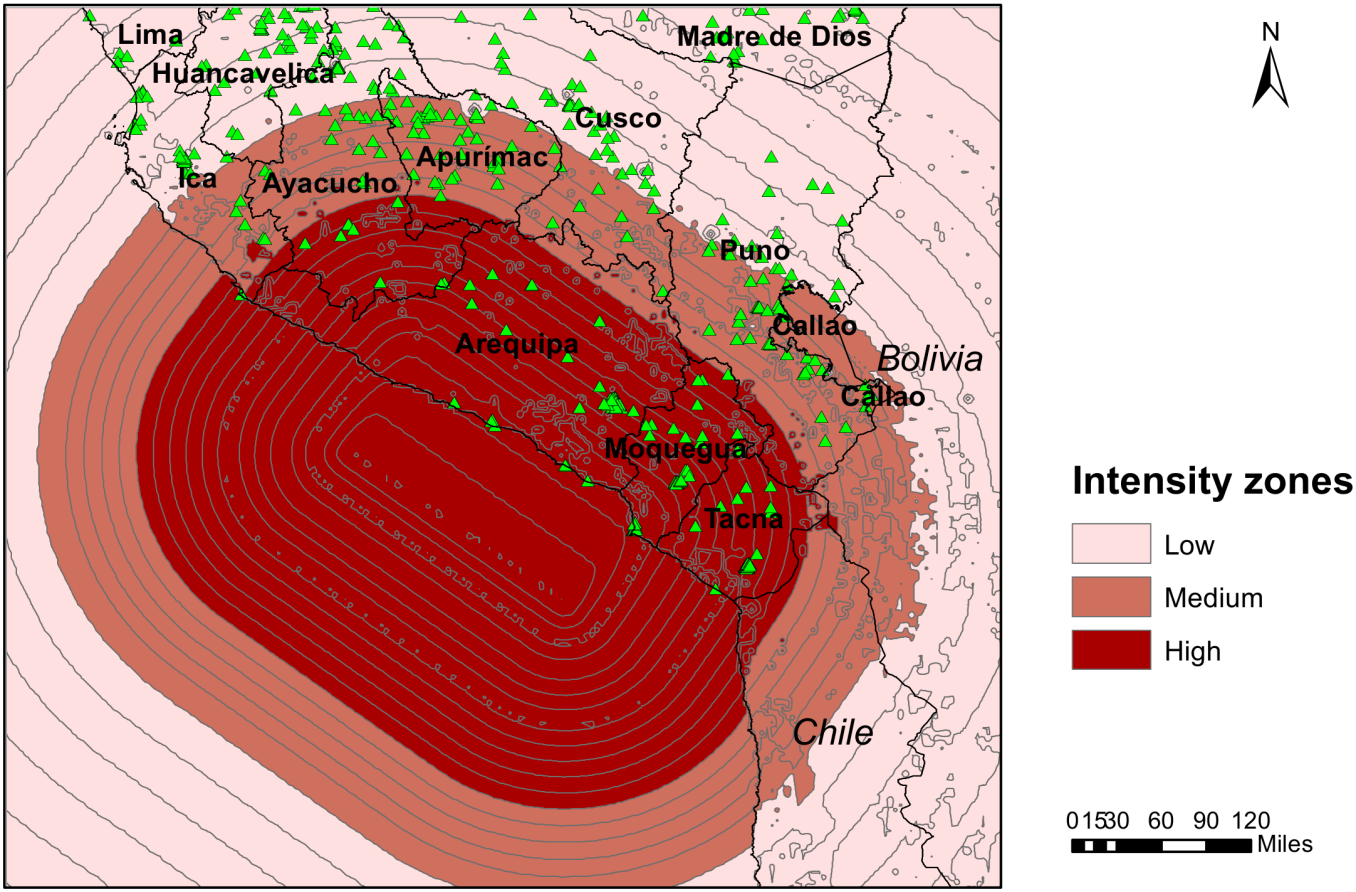
GIS-based map of the areas affected by the 2001 southern Peru earthquake.

Using the results of the spatial join between the ShakeMap and the DHS clusters, we developed a GIS-based map that portrays the earthquake affected southern part of Peru divided into the three intensity zones, with the triangle shaped points marking the locations of the surveyed clusters (Fig 2). This map revealed that the intensity zones cut across administrative borders. For example, the Ayacucho region was exposed to all three levels of intensity. The high and medium intensity zones cover a relatively small area by the coast.

The median and high intensity zones included just over 1,000 children each (Table 1). Over 11,000 of the surveyed children lived in the low intensity zone, corresponding to more than 80% of the total sample. The majority of the children in the sample were born before the earthquake, between years 1995 and 2000.

**Table 1:** Number of children under five years of age, by intensity zone and birth year group.

Birth year group	Low intensity zone	Medium intensity zone	High intensity zone	Total
Born before: 1995–2000	6,144	656	533	7,333
Shortly after: 2001–2003	1,822	223	193	2,238
After: 2004–2008	3,600	381	364	4,345
Total	11,566	1,260	1,090	13,916

The table includes children with a valid height-for-age z-score

We determined that the high intensity zone was mainly urban and the majority of the population was of Spanish ethnicity (Table 2). This zone also had significantly better living standards, higher educational level and better maternal nutritional status (height) than the low and medium intensity zones.

**Table 2:** Pre-earthquake characteristics of households and mothers, by earthquake intensity zone.

	Low exposure (n = 7,066)	Medium exposure (n = 766)	High exposure (n = 712)	
	%	%	%	p-value
***Household characteristics***				
Urbanity	44.4	22.6	72.8	<0.001
Household has electricity	50.6	46.2	77.2	<0.001
***Mother’s characteristics***				
*Ethnicity*				<0.001
Spanish	81.6	38.7	88.5	
Quechua	16.1	54.1	9.7	
Other	2.3	7.2	1.8	
*Education*				<0.001
No Education	9.8	8.6	4.5	
Primary	46.8	58.0	21.1	
Secondary	31.8	26.1	44.9	
Higher	12.6	7.3	29.5	
*Height*				<0.001
<145 cm	17.3	16.1	8.6	

**Fig 3:**
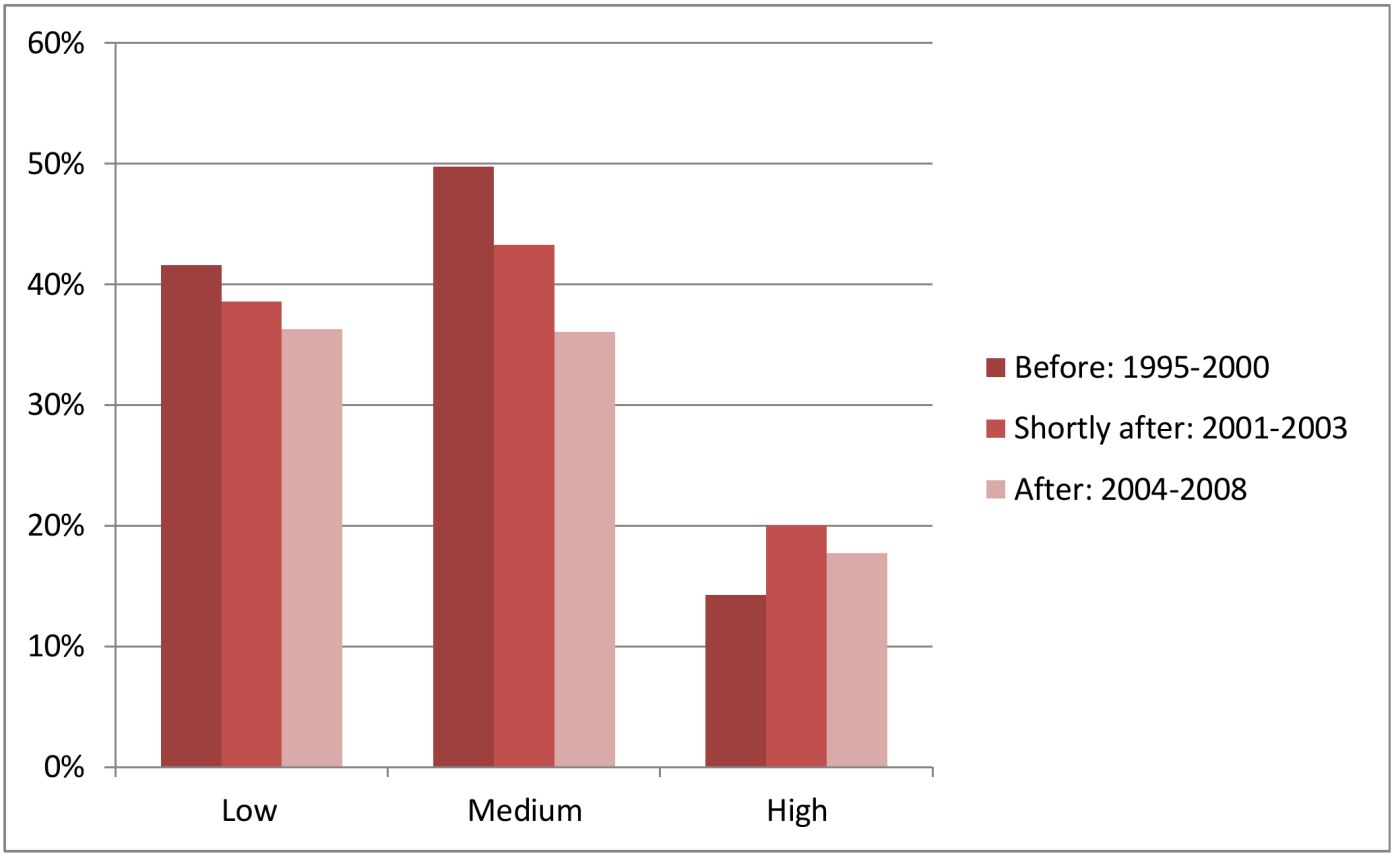
Prevalence of stunting among children 2–4 years of age, by birth year group and intensity zone.

Due to these large pre-earthquake differences in socioeconomic indicators and other important risk factors of stunting, we stratified our analyses by intensity zone. We found that in the high intensity zone 14% of the children born before the earthquake were stunted (Fig 3). The corresponding proportions were 42% in the low intensity zone, and 50% in the medium intensity zone. In the both low and medium intensity zones, stunting prevalence was lower among children born shortly after the earthquake (2001–2003) and even lower in the last birth year group (children born 2004–2008). In the high intensity zone, however, the prevalence increased to 20% for children born shortly after the earthquake. In the last birth year group, the prevalence decreased but remained higher than before the earthquake.

When we investigated the association between birth year group and stunting in the multilevel analysis and adjusted for age of the child, the odds of stunting were not significantly different between any of the birth year groups. The exception was the low intensity zone, in which the odds of stunting among children born several year after the earthquake (2004–2008) was 20% lower than in the pre-earthquake reference group (Table 3, Model 1). However, in the second model, in which we also adjusted for the socioeconomic indicators, children born in the high intensity zone shortly after the earthquake had significantly higher odds of stunting than children born in the same zone before the earthquake (OR 2.01 95% CI 1.03–3.90). For children in the third birth year group, stunting decreased in comparison to the second birth year groups, and its rate was not significantly different from that in the pre-earthquake reference group, suggesting that by this time, sufficient economic and societal recovery had occurred to support healthy development.

**Table 3:** Multilevel regression analysis. Association between birth year group and stunting, by intensity zone. Odds ratios with 95% confidence interval (CI).

	Low exposure			Medium exposure			High exposure		
	No. stunted/ no. not stunted	Model 1^a^ OR (95% CI)	Model 2^b^ OR (95% CI)	No. stunted/ no. not stunted	Model 1^a^ OR (95% CI)	Model 2^b^ OR (95% CI)	No. stunted/ no. not stunted	Model 1^a^ OR (95% CI)	Model 2^b^ OR (95% CI)
Born before: 1995–2000	2143/3713	Referent	Referent	267/370	Referent	Referent	65/445	Referent	Referent
Born shortly after: 2001–2003	697/1115	0.87 (0.74–1.01)	1.08 (0.93–1.26)	99/124	0.93 (0.58–1.51)	1.10 (0.68–1.76)	38/152	1.64 (0.89–3.02)	2.01 (1.03–3.90)
Born after: 2004–2008	1079/2511	0.77 (0.67–0.88)	0.99 (0.86–1.13)	138/243	0.82 (0.54–1.24)	1.05 (0.68–1.60)	49/311	1.11 (0.65–1.91)	1.53 (0.83–2.82)

^a^. Adjusted for age of child

^b^. Adjusted for age of child, educational level of mother and availability of household electricity

## Discussion

### Usefulness of the approach

Our results indicate that long-term public health effects of earthquakes, more specifically effects on childhood stunting, may be evaluated using a combination of ShakeMap and DHS data. Both data sources are readily available online, and may be combined using the geographical coordinates of the surveyed clusters. The repeated measuring-points of DHS surveys allow comparing stunting levels before and after an earthquake, a prerequisite to draw conclusions about its effects. Follow-up data was available for up to seven years after the earthquake, which gives a unique opportunity to study long-term effects. Most previous information about public health effects of earthquakes come from studies with short-term follow up, and/or without baseline data [28,29]. DHS data also included several socio-economic indicators, which allowed us to control for decreasing trends in stunting driven by socioeconomic improvements.

Our work shows that the level of stunting in Peru was elevated among children born after the 2001 earthquake only in areas exposed to high seismic intensity levels. These results are consistent with previous findings indicating that the societal impact of earthquakes swells with increasing levels of intensity [6]. The fact that the level of stunting is lower among children born several years after the earthquake than among those born immediately after the quake, correlates with sufficient time for recovery, both economic and societal, following the disaster.

Our results underline the importance of GIS-based disaster data for assessing effects of a disaster on a given population with high precision. As the intensity levels of the earthquake cut across administrative borders, a division based on administrative boundaries would have resulted in misclassification of earthquake exposure. Our results therefore confirm previous findings that GIS data provide a more precise estimation of disaster affected populations than when administrative borders are used to demarcate disaster zones [15].

We identified a few factors that limit the usefulness of the approach. Firstly, DHS samples are not designated to be broken down into intensity zones. As the sample sizes in the medium and high intensity zones were relatively small in our Peruvian model, they yielded decreased statistical power, especially in the high intensity zone, where stunting prevalence was low compared to the other zones. The same issue has been discussed previously in a Tsunami Recovery Impact Assessment and Monitoring System (TRIAMS) workshop, where the use of national household survey data was recommended to follow the recovery process of the 2004 Indian Ocean tsunami [30]. A suggested solution was to oversample the population in disaster affected areas. Another recommendation was to modify routine health surveys to provide representative statistics of the geographical area affected by the disaster.

Another disadvantage was the large difference between the earthquake intensity zones at “baseline” (i.e. before the earthquake hit). The area most affected by the quake was mainly urban, had relatively high socioeconomic status, and significantly lower levels of stunting than the rest of the country. According to Ukoumunne et al., such baseline imbalance might bias results of effect evaluation studies, because changes over time are dependent on the levels at baseline [31]. For example, the level of stunting might be more likely to decrease in a population with high initial levels, than in a population where levels are already low. This could be an alternative explanation of why stunting decreased only in the low and medium intensity zone. It is, however, less conceivable that this would explain the increased level of stunting in the high intensity zone. Going forward, it will be important to study the effect of baseline imbalance between differently affected populations, as well as to develop analysis methods to account for it.

The cross-sectional design of DHS is another potential weakness of the approach. Large migration from the earthquake-affected areas might change population composition and bias prevalence estimates. For example, if well-off families migrate to a larger extent than poorer families, an artificial increase of stunting would occur in the remaining population. To rule out this possibility, the same children would have to be followed over time in a longitudinal study. To assess the validity of results based on cross-sectional data, it will be important to study patterns of population displacement from affected areas.

### Study limitations

A limitation of our study is that we did not have a gold standard of comparison to know if the approach generated valid results. The lack of previous research also precludes comparing our results to other studies. The social and economic impacts of earthquakes are well-known, at least in the short-term, as is the link between socioeconomic factors and stunting. However, a direct link between earthquakes and stunting has not been reported in previous literature. Nevertheless, the main aim of this study was to test the feasibility of the approach, not to determine the validity of the results.

Our study only evaluated a single earthquake, and some of our assumptions and results may not be applicable to other locales and earthquakes. In addition, the availability of DHS and ShakeMap data differs from case to case. For example, not all DHS surveys collect GPS data, which is necessary to link the respondents to the respective seismic intensity zones. Considering the wealth of both DHS and ShakeMap data, however, it is likely that our approach can be applied to other earthquakes and potentially even other natural disasters, using other sources of GIS disaster data. For example, Guha-Sapir et al. [15] reported on several initiatives that have made GIS-based flood data available for disaster research.

## Conclusions

Long-term public health effects of earthquakes are understudied in disaster research, which may be attributed to the lack of systematically collected health data in affected regions. Our study suggests that a combination of readily available data sources can be used to increase knowledge about such long-term effects. Although limitations exist, given the wealth, easy access, and good quality of DHS and ShakeMap data, it is an approach worth investigating further and considering in future public health and disaster research.

## References

[1] CRED. EM-DAT: The OFDA/CRED International Disaster Database. Brussels: Universitsithealth and disaster re

[2] Doocy S, Daniels A, Packer C, Dick A, Kirsch TD. The human impact of earthquakes: a historical review of events 1980–2009 and systematic literature review. PLoS Curr. 2013;5: 1–39. 10.1371/currents.dis.67bd14fe457f1db0b5433a8ee20fb833 PMC3644288 23857161

[3] Roy N, Thakkar P, Shah H. Developing-world disaster research: present evidence and future priorities. Disaster Med Public Health Prep. 2011;5: 11211 10.1001/dmp.2011.35 21685306

[4] Kahn ME. The Death Toll from Natural Disasters: The Role of Income, Geography, and Institutions. Rev Econ Stat. 2005;87: 271271h Prep. 2011;5: 11211. 10.105.

[5] Carter MR, Little PD, Mogues T, Negatu W. Poverty Traps and Natural Disasters in Ethiopia and Honduras. World Dev. 2007;35: 835–856. 10.1016/j.worlddev.2006.09.010

[6] Fisker PS. Earthquakes and economic growth. Institute for Advanced Development Studies; 2012. Report No.: 01/2012.

[7] Zolala F. Evaluation of the usefulness of maternal mortality ratio for monitoring long-term effects of a disaster: case study on the Bam earthquake. East Mediterr Health J. 2011;17: 976–80. 22355952 10.26719/2011.17.12.976

[8] Black RE, Allen LH, Bhutta ZA, Caulfield LE, de Onis M, Ezzati M, et al Maternal and child undernutrition: global and regional exposures and health consequences. Lancet. 2008/01/22 ed. 2008;371: 243–260. 10.1016/S0140-6736(07)61690-0 18207566

[9] Waterlow JC. Introduction. Causes and mechanisms of linear growth retardation (stunting). Eur J Clin Nutr. 1994/02/01 ed. 1994;48 Suppl 1: S1–4. 8005078

[10] Dong C, Ge P, Ren X, Zhao X, Wang J, Fan H, et al The micronutrient status of children aged 24–60 months living in rural disaster areas one year after the Wenchuan earthquake. PLoS One. 2014;9: 19: 1260. d.1371/journal.pone.0088444 10.1371/journal.pone.0088444 PMC3922868 24533089

[11] Measure DHS. What we do: GPS data collection [Internet]. [cited 7 Jul 2014]. Available: http://dhsprogram.com/What-We-Do/GPS-Data-Collection.cfm

[12] Kawasaki A, Berman ML, Guan W. The growing role of web-based geospatial technology in disaster response and support. Disasters. 2013;37: 201–21. 10.1111/j.1467-7717.2012.01302.x 23278379

[13] Allen TI, Wald DJ, Hotovec AJ, Lin K, Earle PS, Marano KD. An atlas of ShakeMaps for selected global earthquakes U.S. Geological Survey Open-File Report 2008–1236, 35 p.; 2008.

[14] Naghii MR. Public health impact and medical consequences of earthquakes. Rev Panam Salud Publica. 2005/11/05 ed. 2005;18: 216 216 Su15. 16269124 10.1590/s1020-49892005000800013

[15] Guha-Sapir D, Rodriguez-Llanes JM, Jakubicka T. Using disaster footprints, population databases and GIS to overcome persistent problems for human impact assessment in flood events. Nat Hazards. 2011;58: 845–852. 10.1007/s11069-011-9775-y

[16] The World Bank Group. Countries: Peru [Internet]. [cited 13 Jul 2013]. Available: http://data.worldbank.org/country/peru

[17] Central Intelligence Agency. The world factbook: Peru [Internet]. 2013 [cited 12 May 2013]. Available: https://www.cia.gov/library/publications/the-world-factbook/geos/pe.html

[18] National Institute of Statistics and Informatics (INEI). Principales resultados de la Encuesta Nacional de Hogares sobre Condiciones de Vida y Pobreza (ENAHO)-IV Trimestre 2001. 2002.

[19] National Institute of Statistics and Informatics (INEI), United States Agency for International Development (USAID), ICF Macro. Peru DHS, 2007–08—Final Report Continuous (2007–2008) (Spanish). Vasa. Lima, Perú; 2009.

[20] Measure DHS. STATcompiler [Internet]. Calverton: ORC Macro; [cited 28 Apr 2013]. Available: http://www.statcompiler.com/

[21] National Institute of Statistics and Informatics (INEI), United Nations Children’s Fund (UNICEF). Estado de la Niñez Indígena en el Perú. Lima; 2010.

[22] Hammer J, Toledo CZ, Recuay RS. El terremoto del 23 de Junio del 2001 en el Sur del Per PerICEF). Estado dereconocimiento del 27 al 30 de Agosto del 2001 en las zonas afectadas (Tacna, Moquegua y Arequipa) Características generales de las Zonas Afectadas [Internet]. 2001 pp. 1–7. Available: http://www.drmonline.net/drmlibrary/peru.htm#N_1_

[23] National Institute of Statistics and Informatics (INEI), Measure/DHS+ Macro International Inc. Peru DHS, 2000—Final Report (Spanish). Lima, Perú: Measure/DHS+, Macro International Inc., Instituto Nacional de Estadística e Informática Peru; 2001.

[24] National Institute of Statistics and Informatics (INEI), United States Agency for International Development (USAID), Macro International Inc. Peru DHS, 2004–06—Final Report Continuous (2004–2006) (Spanish). Lima, Perú. 2007.

[25] Wald DJ, Worden BC, Pankow KL. ShakeMap® Manual: technical manual, user guide, and software guide. U.S. Geological Survey; 2006.

[26] WHO Multicentre Growth Reference Study Group. WHO Child Growth Standards: Length/height-for-age, weight-for-age, weight-for-length, weight-for-height and body mass index-for-age: Methods and development. Geneva: World Health Organization; 2006.

[27] Victora CG, de Onis M, Hallal PC, Blossner M, Shrimpton R. Worldwide timing of growth faltering: revisiting implications for interventions. Pediatrics. 2010/02/17 ed. 2010;125: e473–80. 10.1542/peds.2009-1519 20156903

[28] Sun J, Huo J, Zhao L, Fu P, Wang J, Huang J, et al The nutritional status of young children and feeding practices two years after the Wenchuan Earthquake in the worst-affected areas in China. Asia Pac J Clin Nutr. 2013;22: 100–8. 10.6133/apjcn.2013.22.1.19 23353617

[29] Polonsky J, Luquero F, Francois G, Rousseau C, Caleo G, Ciglenecki I, et al Public health surveillance after the 2010 haiti earthquake: the experience of mof me worst-affected areas in China. Asia Pac J Clin Nutr. 2013;2rents.dis.6aec18e84816c055b8c2a06456811c7a 10.1371/currents.dis.6aec18e84816c055b8c2a06456811c7a PMC3544554 23330069

[30] United Nations, World Health Organization, International Federation of Red Cross and Red Crescent Societies. Tsunami recovery impact assessment and monitoring system. (TRIAMS) Workshop Bangkok, 3k, 3ac J Clin2006.

[31] Ukoumunne OC, Thompson SG. Analysis of cluster randomized trials with repeated cross-sectional binary measurements. Stat Med. 2001;20: 417 417t 11180311 10.1002/1097-0258(20010215)20:3<417::aid-sim802>3.0.co;2-g

